# Submicroscopic interstitial deletion of chromosome 11q22.3 in a girl with mild mental retardation and facial dysmorphism: Case report

**DOI:** 10.1186/1755-8166-4-17

**Published:** 2011-08-22

**Authors:** Danijela Krgovic, Natasa Marcun Varda, Andreja Zagorac, Nadja Kokalj-Vokac

**Affiliations:** 1 Laboratory of Medical Genetics, Maribor University Medical Centre, Maribor, Slovenia; 2 Maribor Medical Faculty, University of Maribor, Maribor, Slovenia

**Keywords:** 11q22.3 deletion, mild mental retardation, facial dysmorphism

## Abstract

**Background:**

Except for terminal deletions that lead to Jacobsen syndrome, interstitial deletions involving the long arm of chromosome 11 are not frequently reported. A clinically distinct phenotype is usually observed in these cases, and no clear genotype-phenotype correlation is proposed.

**Results:**

Here we present a case study of a 5-year-old girl with *de novo *submicroscopic deletion of chromosome 11q22.3 with mild mental retardation and facial dysmorphism. A standard cytogenetic analysis did not reveal any structural aberrations. In contrary, array-CGH analysis indicated a small deletion of 11q22.3.

**Discussion:**

To our knowledge, this is the smallest 11q22.3 deletion reported in literature, containing nine RefSeq genes. Although none of the deleted genes are obvious candidates for the features observed in our patient, genes *CUL5 *and *SLN *could play a key role in the features described.

## Background

Interstitial deletions of the long arm of chromosome 11 are not often reported in literature. Usually, the terminal deletions that cause Jacobsen syndrome are well-described, whereas interstitial aberrations are less well-defined due to the band pattern similarity in this region and limited resolution offered by classical cytogenetic methods [1,2].

The clinical phenotype in 11q deletion patients usually varies depending on the size and position of the deletion. The same trend can be observed when 11q22.3 deletions are compared. No clear genotype-phenotype correlation can be observed, mainly because the deletions encompassing this region also include other bands [1-12]. The most common phenotypic features observed in these patients are mild to severe mental retardation, developmental delay, a high arched or cleft palate (in some cases with a cleft lip), and hypertelorism. A delay in speech and walking are usually present in all cases [1,2,8,10,11]. Additional features such as trigonocephaly [10,11], small hands and feet [8,11], syndactyly [1], simian crease [2,10], and others are also observed. To the best of our knowledge a case with a deletion limited to 11q22.3 has so far not been reported.

Here we present a girl with *de novo *submicroscopic deletion of 11q22.3, detected by array-CGH analysis (aCGH). Despite the fact that only much larger deletions have been described previously, we propose a genotype-phenotype correlation for this region. Hopefully, the discovery of new patients with microdeletions in 11q22.3 will lead to a better understanding of the roles that the deleted genes play in the clinical outcome.

## Case presentation

**Figure 1:**
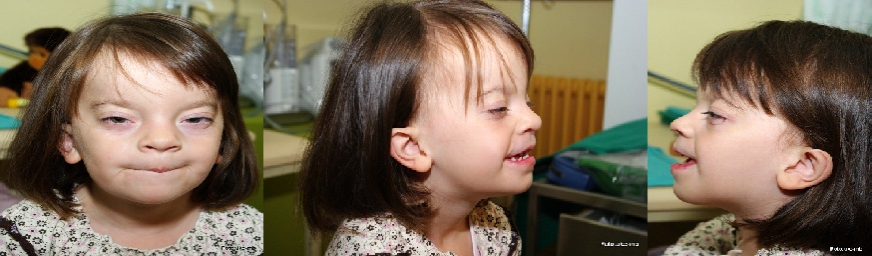
**The facial features observed in our patient include ptosis, hypertelorism, low-set dysplastic ears and a prominent forehead**.

An infant girl was born at 36 weeks gestation to a healthy 23-year-old mother and 28-year-old father. Her birth weight was 2690 g (50^th ^centile for gestational age), her birth length was 47 cm (25^th ^centile), her head circumference was 32.5 cm (25^th ^centile) and her Apgar score was 9/9. After the birth there were no major concerns, although patent ductus arteriosus and haemodynamically significant atrial septal defect (ASD) had been diagnosed, and pronounced dysmorphic features had been observed. She had upward and slanted palpebral fissures with ptosis of both. This was more pronounced in the left upper palpable. Hypertelorism was also observed and the nose was small in size and saddle-shaped. She had a prominent frontal part of the skull, giving the false impression of macrocrania. Her ears were low-set, asymmetrical, triangular and slightly protuberant. Both lips were accentuated in shape and sometimes stretched in an asymmetrical manner (Figure 1). A high-arched palate was also observed. There was evidence of general muscular hypotonia and her developmental milestones were delayed. She sat up without help at 12 months and started walking at 18 months. Her speech development was also late, and only developed after she was three years old. Her social and psychical development were surprisingly good. A magnetic resonance imaging (MRI) scan of the brain showed hyperintensive changes in the white matter and hypoplastic corpus callosum. The electroencephalogram (EEG) was normal and there were no convulsions.

At 12 months, she suffered from serious acute pyelonephritis, and bilateral vesicoureteral reflux (VUR) was diagnosed thereafter. The ultrasound kidney volumes were normal with no major dilatation of the renal pyelon. The endoscopic treatment of the VUR was performed on two occasions. Lastly, after classical surgical treatment, the VUR was successfully treated. At that time, her neurodevelopment was equivalent to that of a 9-month-old infant.

Assessments of the child are being carried out every six months. With the exception of one case of pneumonia and one case of acute laryngitis, no additional health problems had been reported. There were no recurrent urinary tract infections.

The girl is currently 5 years old. She weighs 15.5 kg (10^th ^centile), has a height of 110 cm (50^th ^centile) and a head circumference of 49 cm (10^th ^centile). The dysmorphic features are still prominent. She has lower basic muscle tone, especially in the shoulder muscles and the pelvic girdle. Her muscle strength is fairly good and her reflexes are normal. There are no major problems with fine motorics and there are no abnormal movements or contractures. Her palms, especially hypothenar, are hypoplastic and her fingers are thin. Her foot arches are prominent. Hips incline to internal rotation with knees adduction and inward feet rotation, which has no major impact on her gait. Both renal function and blood pressure are normal. At present, she is preparing for the correction of a congenital heart defect.

She is managed with a team of experts and receives special logopaedic, psychological, and defectological assistance. The girl is motivated, cooperates and strives for success and progress. She is pleasant, friendly, good-natured and no emotional or behavioural problems have been observed. Despite her relatively normal development, a psychological examination (Goodenough Draw, Gessell Drawing Test, Brunet-Lesine scale, Vineland Social Maturity Scale, Wechsler Intelligence Scale for Children - WISC) reveals some cognition deficits and borderline intellectual functioning, requiring specialised help and causing delays in her formal education. She follows and understands simple instructions, speaks in sentences, but her pronunciation is unclear. Her global intelligence quotient is 76 (WISC test). Her social maturity meets the requirements of a 5-year-old child (Vineland Social Maturity Scale). According to the Brunet-Lezine scale, her mental age is that of a 4.6 year old child.

## Results

**Figure 2:**
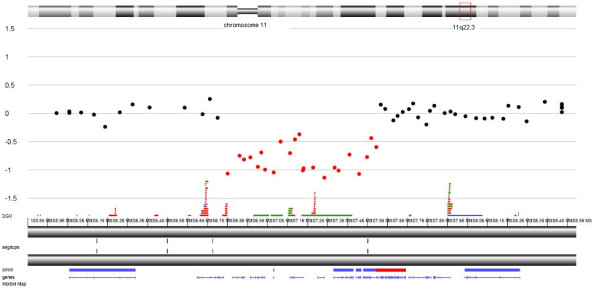
**An aCGH analysis of the patient revealed a deletion of the 11q22.3 region**. The breakpoints of the deletion were therefore determined to be arr 11q22.3(107349817-108093259)x1 dn (hg19; NCBI build 37).

**Figure 3:**
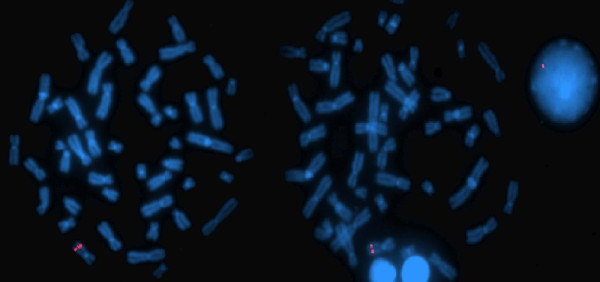
**A FISH analysis of the patient was carried out using BAC probe RP11-56J3 (labelled red) encompassing the 11q22.3 region**. A single red signal can be seen on the normal chromosome 11 and in the nucleus, whereas the signal is absent in the chromosome with the 11q22.3 deletion.

As conventional karyotyping of the proband did not reveal any structural rearrangements, the karyotype was therefore determined to be 46, XX. Molecular karyotyping was performed on the Agilent 60K platform. Data analyses indicated a 743 kb deletion of 11q22.3 (Figure 2). An aCGH analysis of the patients' parents did not show any abnormalities; thus deletion has been determined to be *de novo*. Confirmation of the deletion was performed by fluorescent *in situ *hybridisation (FISH) using BAC probe RP11-56J3. A single red signal can be seen on the normal chromosome 11 and in the nucleus, whereas the signal is absent in the chromosome with the 11q22.3 deletion (Figure 3).

## Discussion

In the relevant literature, deletions involving the long arm of chromosome 11 are usually associated with Jacobsen syndrome, a condition with multiple anomalies caused by the terminal deletion of this chromosome [13]. In contrast, interstitial deletions of 11q are less common and often not fine-mapped, due to the similarity between two band patterns (11q14 and 11q22) when conventional karyotyping is performed [1,2]. Furthermore, no clear genotype-phenotype correlations have been proposed in reported patients. Here, we present a new patient with *de novo *submicroscopic deletion of chromosome 11q22.3. The deletion was determined by aCGH (Figure 2) and confirmed by a FISH analysis (Figure 3). The clinical phenotype in our patient includes mild mental, developmental and speech delay, without emotional and behavioural difficulties. She has prominent facial dysmorphisms, ptosis, hypertelorism, low-set dysplastic ears and a prominent forehead (Figure 1). Hypoplastic corpus callosum, VUR and ASD were also diagnosed.

Much larger deletions, overlapping this region, have been described previously. To our knowledge, none of the reported cases had deletion limited only to 11q22.3; therefore, this is the smallest deletion of 11q22.3 reported in the literature. Most of the patients with an overlapping deletion of this region had mild to severe mental retardation and developmental delay, usually depending on the size and position of the deletion. A high arched or cleft palate was observed in all cases. Dysmorphic features such as trigonocephaly, microcephaly, hypertelorism, eye anomalies, hypotonia, speech delay and others were also observed [1-12].

Apart from unspecific features, such as mental and developmental delay, and eye and palate anomalies, no specific common features were observed in our case study or those previously described. Interestingly, the Decipher patient 950 with a small inherited duplication of this region (arr 11q22.3(107792125-107953547)x3 pat (hg19; NCBI build 37)) has concordant phenotypic features with our patient, including strabismus, kidney anomalies, and hypoplastic corpus callosum, all of which have not been observed in other cases.

The deletion interval in our case contains nine RefSeq genes: *ALKBH8, ELMOD1, LOC643923, SLN, SLC35F2, RAB39, CUL5, ACAT1 *and *NPAT*. The *ALKBH8 *gene encodes the ALKBH8 protein, which belongs to a group of human homologues of AlkB protein in *E. coli*. ALKBH8 is involved in the repair of methylation-induced DNA damage by oxidative demethylation [14]. Its role was described in the prevention of mutation and carcinogenesis in various types of mammalian cells [15,16]. Secondly, the *ELMOD1 *gene encodes a GTPase-activating protein. As ELMOD2 protein, another member of ELMO family, ELMOD1 acts on the Arf family, which are members of the Ras superfamily of proteins [17]. The *LOC643923 *gene encodes a hypothetical protein. Another gene, *SLN*, encodes a small proteolipid that catalyses the ATP-dependent transport of Ca(2+) from the cytosol into the lumen of the sarcoplasmic reticulum in muscle cells [18]. Although, *Sln*-null mice do not exhibit any cardiac anomalies, a study from Babu et al., 2007, demonstrated that homozygous-null mice exhibit increased cardiac contractility [19]. The *SLC35F2 *gene encodes a member of nucleotide sugar transporters in the Golgi apparatus and the endoplasmic reticulum [20]. A member of the Ras-like family, involved in vesicle budding, docking, and fusion in endocytosis and exocytosis pathways, is encoded by the *RAB39 *gene [21-24]. The *CUL5 *gene encodes vasopressin-activated calcium-mobilising receptor 1, which is a component of a multiple SCF-like ECS E3 ubiquitin-protein ligase complex. This complex mediates the ubiquitination and subsequent proteasomal degradation of target proteins [25,26]. The enzyme encoded by the *ACAT1 *gene is involved in the metabolism of acetyl-CoA. An autosomal recessive beta-ketothiolase deficiency is described as an inborn error of isoleucine catabolism caused by the disruption of this gene [27]. The last, *NPAT *gene, encodes the nuclear protein of the ataxia telangiectasia mutated locus. This is a housekeeping gene involved in the cell cycle. Imai et al., 1997, proposed that the *NPAT *gene shares the same promoter region with the *ATM *gene, the disruption of which causes an autosomal recessive ataxia telangiectasia. A loss of heterozygosity of this gene along with others on 11q22-q24 locus has been frequently described in various types of tumours [28].

Although none of the genes described previously indicate their direct role in the development of phenotype seen in our patient, Feng et al., 2007, reported that the *CUL5 *gene plays an essential role in neuron migration during cortical development [29]. The haploinsufficiency of this gene has not yet been reported in patients or knockout mice; therefore, a direct genotype-phenotype correlation is not possible and further investigation should be conducted. A lower expression of the *SLN *gene was described in children with congenital heart defects by Vittoius et al., 2007, but not in the association with ASD [30].

## Conclusion

The majority of the patients reported previously with the deletion containing the 11q22.3 region display mental retardation and facial dysmorphism, including the patient presented here. Specific features such as strabismus, kidney anomalies, and hypoplastic corpus callosum are observed only in Decipher patient 950 and our patient. Both of these probands have aberrations which are limited only to 11q22.3.

None of the deleted genes are obvious candidates for the features observed in our patient, although genes *CUL5 *and *SLN *could play a key role in the features described. The scarcity of patients with 11q22.3 microdeletions aggravates a clear genotype-phenotype correlation. Hopefully, the discovery of new patients with microdeletions in this region will lead to a better understanding of the roles that the deleted genes play in the clinical outcome.

## Materials and methods

### Chromosome analysis

The chromosomal analysis was performed on metaphase chromosomes using peripheral blood lymphocytes. The chromosomes were harvested and analysed in accordance with standard cytogenetic methods.

### Molecular karyotyping

Molecular karyotyping was performed on DNA extracted from peripheral blood leukocytes of both, the patient and her parents. An aCGH analysis was performed using Agilent SurePrint G3 Human CGH Microarray Kit 8 × 60K (Agilent Technologies, Santa Clara, CA, USA). The assay was performed according to the manufacturers' instructions with minor modifications. The obtained data was analysed using the arrayCGHbase software tool [31]. The DECIPHER [32] and ISCA [33] databases were used for the genotype-phenotype correlations.

### FISH analysis

The fluorescent *in situ *hybridisation (FISH) analysis was performed using BAC probe RP11-56J3. The BAC clone was selected from the human library RPCI-11 according to the UCSC Human Genome Assembly (March 2006 release) [34].

### Consent

Written informed consent was obtained from the parents of the patient for publication of this case report and accompanying images. A copy of written consent is available for review by the Editor-in-Chief of this journal.

## Competing interests

The authors declare that they have no competing interests.

## Authors' contributions

DK wrote the manuscript and carried out the molecular karyotyping and data analysis; NMV performed a clinical analysis of the patient; AZ conducted the cytogenetic analysis; and NKV coordinated the study. All the authors have read and approved the manuscript.
